# Evaluation of ChatGPT's performance in providing treatment recommendations for pediatric diseases

**DOI:** 10.1002/pdi3.42

**Published:** 2023-11-20

**Authors:** Qiuhong Wei, Yanqin Wang, Zhengxiong Yao, Ying Cui, Bo Wei, Tingyu Li, Ximing Xu

**Affiliations:** ^1^ Children Nutrition Research Center Children's Hospital of Chongqing Medical University National Clinical Research Center for Child Health and Disorders Ministry of Education Key Laboratory of Child Development and Disorders China International Science and Technology Cooperation Base of Child Development and Critical Disorders Chongqing Key Laboratory of Childhood Nutrition and Health Chongqing China; ^2^ College of Medical Informatics Medical Data Science Academy Chongqing Engineering Research Center for Clinical Big‐Data and Drug Evaluation Chongqing Medical University Chongqing China; ^3^ Department of Nephrology Children's Hospital of Chongqing Medical University Chongqing China; ^4^ Department of Neurology Children's Hospital of Chongqing Medical University Chongqing China; ^5^ Department of Biomedical Data Science Stanford University School of Medicine Stanford California USA; ^6^ Department of Global Statistics and Data Science BeiGene USA Inc. San Mateo California USA; ^7^ Big Data Center for Children's Medical Care Children's Hospital of Chongqing Medical University Chongqing China

**Keywords:** ChatGPT, pediatrics disease, treatment

## Abstract

With the advance of artificial intelligence technology, large language models such as ChatGPT are drawing substantial interest in the healthcare field. A growing body of research has evaluated ChatGPT's performance in various medical departments, yet its potential in pediatrics remains under‐studied. In this study, we presented ChatGPT with a total of 4160 clinical consultation questions in both English and Chinese, covering 104 pediatric conditions, and repeated each question independently 10 times to assess the accuracy of its responses in pediatric disease treatment recommendations. ChatGPT achieved an overall accuracy of 82.2% (95% CI: 81.0%–83.4%), with superior performance in addressing common diseases (84.4%, 95% CI: 83.2%–85.7%), offering general treatment advice (83.5%, 95% CI: 81.9%–85.1%), and responding in English (93.0%, 95% CI: 91.9%–94.1%). However, it was prone to errors in disease definitions, medications, and surgical treatment. In conclusion, while ChatGPT shows promise in pediatric treatment recommendations with notable accuracy, cautious optimism is warranted regarding the potential application of large language models in enhancing patient care.

## Graphical abstract

This study evaluated ChatGPT's ability to provide pediatric treatment recommendations using 4160 clinical consultation questions, covering 104 conditions. Overall, ChatGPT achieved an accuracy of 82.2%, with strengths in common diseases and general treatment advice, particularly in English. However, errors were common in disease definitions, medications, and surgical treatment recommendations.

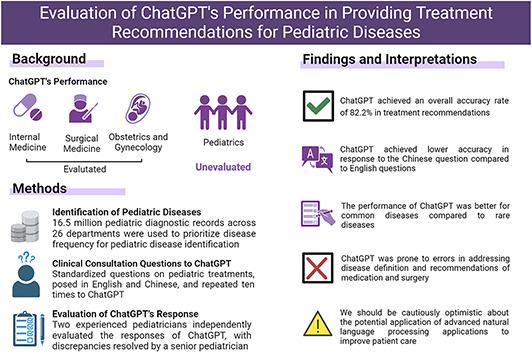

## 1 INTRODUCTION

With the rapid advancement of artificial intelligence technology, natural language processing models have become increasingly prevalent. ChatGPT, a general‐purpose artificial intelligence language model with over 100 million users, has been widely applied to various fields.^1^ In the medical field, ChatGPT has passed the United States Medical Licensing Examination but failed the Chinese equivalent.^2^, ^3^ Growing interest surrounds ChatGPT's potential to assist physicians in clinical medicine.^4^, ^5^

An expanding body of research continues to evaluate ChatGPT's performance in various medical applications.^6^ An early attempt to use ChatGPT as a clinical diagnostic tool has demonstrated modest success, with algorithm performance remaining lower than that of practicing physicians.^7^ Subsequent investigations have assessed the efficacy of the model across several medical disciplines. In internal medicine, ChatGPT has proven capable of answering common patient questions about colonoscopies,^8^ providing advice on cardiovascular disease prevention,^9^ suggesting appropriate antimicrobial usage for infectious diseases,^10^ and participating in the American College of Gastroenterology Self‐Assessment Test.^11^ Moreover, ChatGPT's responses have been benchmarked against physicians' responses to patient inquiries.^12^ In the field of surgery, ChatGPT has the potential to provide prevention and screening recommendations for breast cancer,^13^ addresses questions and concerns from patients with prostate cancer,^14^ and assists in the surveillance and diagnosis of liver cancer.^15^ In the area of obstetrics and gynecology, the AI model has even been involved in clinical examinations and has provided fertility counseling.^16^, ^17^

However, only a limited number of studies have evaluated the performance of ChatGPT in pediatric diseases. To bridge this gap, we presented ChatGPT with 4160 clinical consultation questions, covering 104 pediatric conditions in both English and Chinese. Each question was independently repeated 10 times to evaluate the accuracy of ChatGPT's responses in pediatric disease treatment recommendations.

## 2 MATERIALS AND METHODS

### 2.1 Identification of pediatric diseases

As opposed to relying solely on literature or individual experiences, this study utilized the Big Data Center database of the Children's Hospital of Chongqing Medical University to ensure an objective and comprehensive disease selection. Relying solely on literature or experiences might introduce biases and potentially miss emerging trends in pediatric diseases. This study leveraged real‐world medical records, providing a more comprehensive representation of pediatric diseases as observed in our clinical practice.

Utilizing the database from the Big Data Center, a total of 16,592,422 diagnostic records were collected for children who visited the hospital between January 2018 and December 2022. By analyzing electronic medical records from 26 clinical departments over the past 5 years, we ranked diseases based on their frequency. For each department, we chose four diseases: the top three common diagnoses and the most frequent rare disease. The definition of a “rare disease” is one with a prevalence rate of <1/10,000, consistent with the “Chinese Rare Disease Catalog.”^18^ The patient records from the database were only used for the selection of diseases without utilizing them to generate study cases. The clinical departments included immunology, infectious diseases, gastroenterology, respiratory medicine, rehabilitation, endocrinology, neurology, nephrology, neonatology, cardiovascular medicine, hematology, hepatobiliary surgery, orthopedics, urology, neonatal surgery, burns and plastic surgery, neurosurgery, gastrointestinal surgery, cardiothoracic surgery, oncology surgery, otolaryngology, stomatology, dermatology, child health care, psychology, and ophthalmology.

### 2.2 Clinical consultation questions to ChatGPT

Inputting original clinical consultations from health care systems into an online chatbot raised potential ethical and data security concerns.^12^ De‐identification of patient information, required to comply with ethical guidelines, could alter the context and consequently affect the chatbot's responses. To mitigate these concerns, we utilized simulated clinical scenarios created by pediatricians, culminating in the design of open‐ended clinical consultation questions. The questions adhered to a similar framework for all diseases, beginning with a general inquiry, such as “What is the treatment for a child diagnosed with Graves' disease?” followed by a more specific question containing detailed information, such as “The patient is a 5‐year‐old child weighing 20 kg diagnosed with Graves' disease. What is the preferred medication and dosage for this patient?” The comprehensive list of questions posed to ChatGPT is displayed in Table S1. To ensure the accuracy of the chatbot's responses, standardized clinical consultation questions were presented to ChatGPT (version GPT‐4, OpenAI) via a web interface between April 5 and April 12, 2023. In line with previous studies, we presented a single‐round question that ChatGPT could address in a single interaction without the need for additional clarifying questions.^12^, ^19^ To consider the randomness of ChatGPT's responses and to assess the model's performance across diverse linguistic contexts, each question was repeated 10 times in 10 new chats, in both English and Chinese.

### 2.3 Evaluation of ChatGPT's response

Two pediatricians from the Children's Hospital of Chongqing Medical University, each with over 5 years of practical experience and having completed standardized training across all pediatric subspecialties (including neurology, cardiology, and nephrology among 16 other departments), independently evaluated ChatGPT's responses. The assessments were conducted in accordance with the most recent version of clinical treatment guidelines. In cases of discrepancy, a third adjudicator, a senior pediatrician with over 15 years of practical experience, was consulted. Following the method set forth by Duong et al.^20^ and Hirosawa et al.,^21^ the evaluation of ChatGPT's responses was binary, categorized as either correct or incorrect, with a detailed description of the error type provided (Figure 2).

### 2.4 Statistical analysis

Statistical analyses were conducted using R 4.1.0. Accuracy rates were expressed as frequencies (percentages) with 95% confidence intervals (CIs) based on an approximately normal distribution. A mixed‐effect model was used to analyze the relationship between the number of correct responses and various factors. In this model, the fixed effects included disease prevalence (common/rare diseases), language (English/Chinese), and question type (general/specific), and the repeated measurements are considered as random effects. Odds ratios (ORs) and their corresponding 95% CIs were calculated to indicate the strength and direction of the associations. Statistical significance was determined using a significance level of *p* value < 0.05.

## 3 RESULTS

**FIGURE 1:**
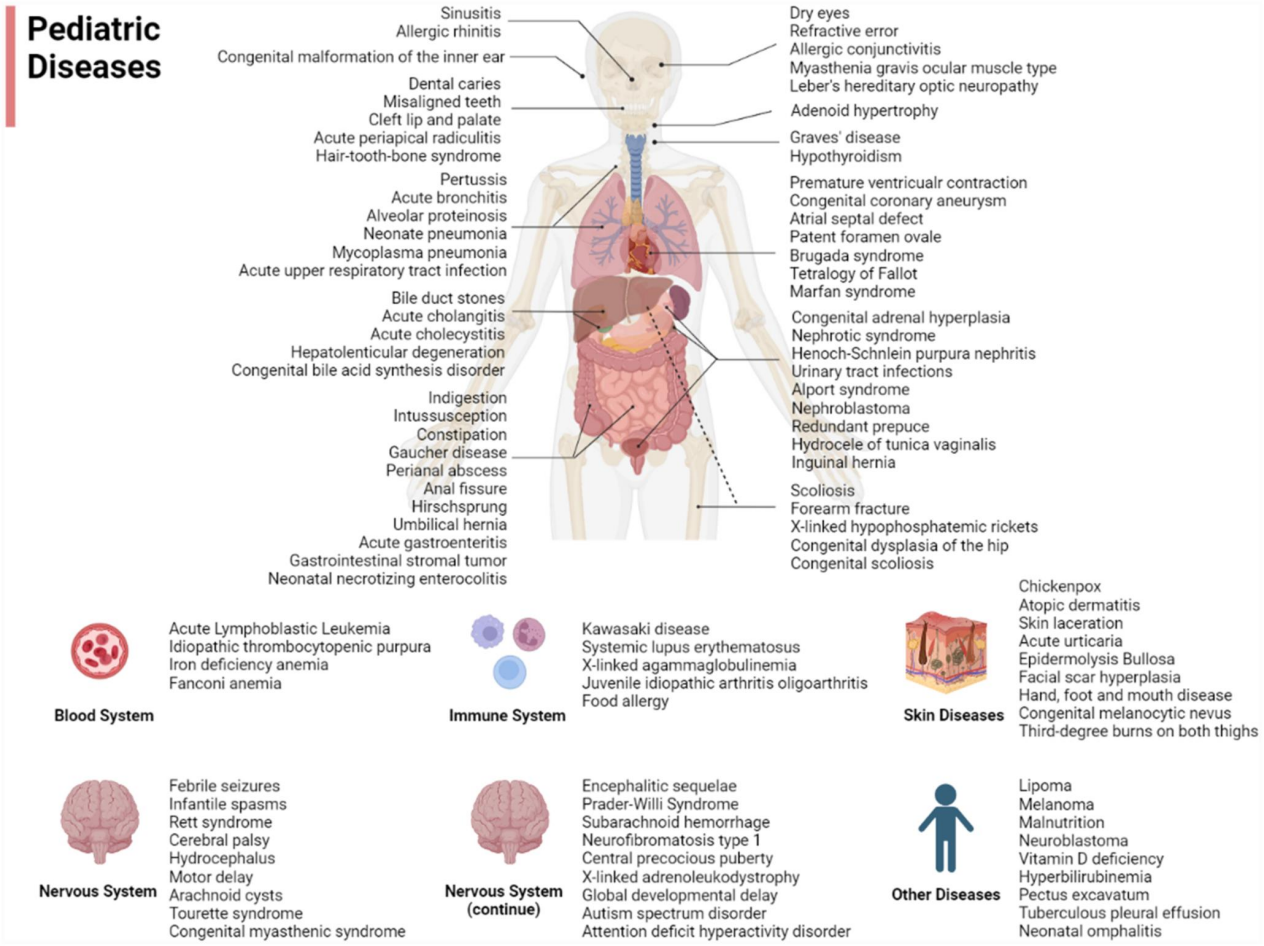
The 104 pediatric diseases selected for analysis of ChatGPT's performance in treatment recommendation.

This study comprehensively assessed ChatGPT's recommendations for pediatric diseases. To achieve this, 104 pediatric diseases were identified, encompassing all anatomical systems in children and including both common and rare conditions (Figure 1). For each disease, ChatGPT was posed with two clinical consultation questions about treatment, including general treatment principles as well as specific treatments such as medication dosages. All questions were repeated independently 10 times, in both Chinese and English, resulting in a total of 104 × (10 × 2 + 10 × 2) = 4160 questions. The responses provided by ChatGPT to these questions were independently evaluated for correctness by two pediatricians. The percentage of inter‐rater agreement between the two evaluators was 93.5%. The remaining 6.5% of ChatGPT's responses were reviewed and discussed with a third senior pediatrician in order to reach a consensus.

We found that ChatGPT correctly answered 3419 questions, achieving an overall accuracy of 82.2% (95% CI: 81.0%–83.4%). The performance varied as follows: 71.4% (1485/2080, 95% CI: 69.5%–73.3%) for Chinese questions, 93.0% (1934/2080, 95% CI: 91.9%–94.1%) for English questions, 84.4% (2634/3120, 95% CI: 83.2%–85.7%) for common diseases, 75.5% (785/1040, 95% CI: 72.9%–78.1%) for rare diseases, 83.5% (1736/2080, 95% CI: 81.9%–85.1%) for general questions, and 80.9% (1683/2080, 95% CI: 79.2%–82.6%) for specific questions. Table 1 shows the frequency distributions of ChatGPT's correct responses over 10 repeated questions. The table further breaks down the distribution by language and differentiates between common/rare diseases and general/specific questions. For example, in the context of general questions posed in English, ChatGPT answered correctly in all 10 instances of repeated queries, whereas it displayed a 33.7% accuracy rate for questions presented in Chinese.

**TABLE 1:** Frequency distribution of ChatGPT's correct responses across 10 repeated inquiries (*N*, %).

Number of correct responses	0	1	2	3	4	5	6	7	8	9	10
English 1 (*N* = 104)	0 (0%)	0 (0%)	1 (1.0%)	2 (1.9%)	0 (0%)	2 (1.9%)	3 (2.9%)	0 (0%)	1 (1.0%)	7 (6.7%)	88 (84.6%)
Common diseases (*N* = 88)	0 (0%)	0 (0%)	1 (1.3%)	1 (1.3%)	0 (0%)	1 (1.3%)	1 (1.3%)	0 (0%)	1 (1.3%)	4 (5.1%)	69 (88.5%)
Rare diseases (*N* = 26)	0 (0%)	0 (0%)	0 (0%)	1 (3.8%)	0 (0%)	1 (3.8%)	2 (7.7%)	0 (0%)	0 (0%)	3 (11.5%)	19 (73.1%)
English 2 (*N* = 104)	1 (1.0%)	1 (1%)	0 (0%)	0 (0%)	2 (1.9%)	4 (3.8%)	2 (1.9%)	1 (1%)	8 (7.7%)	15 (14.4%)	70 (67.3%)
Common diseases (*N* = 88)	0 (0%)	1 (1.3%)	0 (0%)	0 (0%)	2 (2.6%)	3 (3.8%)	2 (2.6%)	1 (1.3%)	6 (7.7%)	8 (10.3%)	55 (70.5%)
Rare diseases (*N* = 26)	1 (3.8%)	0 (0%)	0 (0%)	0 (0%)	0 (0%)	1 (3.8%)	0 (0%)	0 (0%)	2 (7.7%)	7 (26.9%)	15 (57.7%)
Chinese 1 (*N* = 104)	7 (6.7%)	4 (3.8%)	3 (2.9%)	1 (1%)	2 (1.9%)	7 (6.7%)	9 (8.7%)	8 (7.7%)	13 (12.5%)	15 (14.4%)	35 (33.7%)
Common diseases (*N* = 88)	4 (5.1%)	0 (0%)	2 (2.6%)	1 (1.3%)	2 (2.6%)	5 (6.4%)	5 (6.4%)	7 (9%)	11 (14.1%)	12 (15.4%)	29 (37.2%)
Rare diseases (*N* = 26)	3 (11.5%)	4 (15.4%)	1 (3.8%)	0 (0%)	0 (0%)	2 (7.7%)	4 (15.4%)	1 (3.8%)	2 (7.7%)	3 (11.5%)	6 (23.1%)
Chinese 2 (*N* = 104)	6 (5.8%)	5 (4.8%)	2 (1.9%)	7 (6.7%)	5 (4.8%)	4 (3.8%)	4 (3.8%)	8 (7.7%)	16 (15.4%)	9 (8.7%)	38 (36.5%)
Common diseases (*N* = 88)	3 (3.8%)	3 (3.8%)	1 (1.3%)	5 (6.4%)	5 (6.4%)	3 (3.8%)	3 (3.8%)	8 (10.3%)	11 (14.1%)	6 (7.7%)	30 (38.5%)
Rare diseases (*N* = 26)	3 (11.5%)	2 (7.7%)	1 (3.8%)	2 (7.7%)	0 (0%)	1 (3.8%)	1 (3.8%)	0 (0%)	5 (19.2%)	3 (11.5%)	8 (30.8%)

*Note*: The column labeled “0” indicates ChatGPT provided 0 correct answer across all 10 repeat times. Similarly, the column labeled “1” represents ChatGPT answered correctly once and so forth.

Abbreviations: Chinese 1, general question in Chinese; Chinese 2, specific question in Chinese; English 1, general question in English; English 2, specific question in English; *N*, number of diseases.

Furthermore, a mixed effect model was performed to examine the relationship between the likelihood of providing a correct response (outcome) and factors including disease prevalence (common/rare diseases), language (English/Chinese), and question type (general/specific). Our findings revealed an OR of 1.86 (95% CI: 1.56–2.23) for common diseases compared to rare diseases, suggesting that ChatGPT was 1.86 times more likely to provide a correct response for common diseases. Similarly, the OR was 5.42 (95% CI: 4.46–6.59) for questions in English compared to those in Chinese, indicating a higher likelihood of correct responses for English questions. Lastly, an OR of 1.21 (95% CI: 1.03–1.43) was observed for general questions compared to specific questions.

**FIGURE 2:**
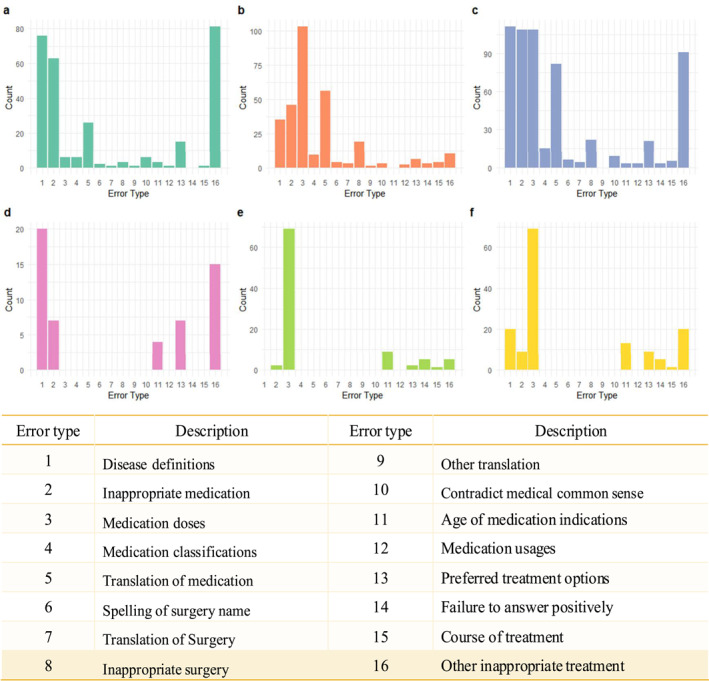
Error analysis of ChatGPT's responses. (A) General questions in Chinese; (B) specific questions in Chinese; (C) general and specific questions in Chinese; (D) general questions in English; (E) specific questions in English; (F) general and specific questions in English. Translation means translation of the words from English to Chinese.

To provide a more detailed assessment of ChatGPT's performance, we conducted an analysis of the errors made by ChatGPT (Figure 2). In total, 16 types of errors were identified, primarily involving disease definitions, medications, and surgical processes. Table S2 showcases 16 selected dialogs that exemplify all identified types of incorrect responses from ChatGPT. In particular, incorrect disease definitions, such as the faulty explanation that “Central precocious puberty refers to the state of sexual maturity of children in girls under 6 years of age and boys under 8 years of age” while the correct age is 8 for girls and 9 for boys. Medication‐related errors encompassed several categories, including inappropriate medication recommendations such as Tetrabenazine for Tourette syndrome (which is actually used to treat Huntington's disease), misclassification of medication such as citing Tetracaine as an antihistamine, incorrect dosages such as the proposed treatment for infantile spasms: Adrenocorticotropic hormone at a dose of 0.15–0.5 mg/kg orally twice daily (the correct dose is 1–2 U/kg/day), inaccurate translations of drug names such as “amoxicillin (Strattera),” and errors in medication usage, age indications, and courses of treatment. Surgery‐related errors involved incorrect spellings of surgery names, the mistranslation of surgery names, and recommending inappropriate surgeries, such as “laparoscopic surgeries for perianal abscess.” Other errors included contradictions to medical common sense such as “massages can promote lymphatic drainage,” inappropriate treatments such as “performing craniotomy to treat congenital myasthenia gravis syndrome,” failures to answer positively such as “I am an AI, I can't provide you with medical advice.”

Notably, a higher number of errors were observed in the Chinese questions compared to the English questions. The major errors in Chinese included disease definitions, inappropriate medication, medication dose, translation of medication, and inappropriate treatment. In contrast, errors in responses to English questions were mainly related to medication doses.

## 4 DISCUSSION

This study provides a comprehensive evaluation of ChatGPT's performance in providing treatment recommendations for pediatric diseases. The results indicated that ChatGPT achieved an overall accuracy rate of 82.2%, suggesting its potential application as a supportive tool in pediatric clinical practice and holding promise to unlock untapped productivity so that pediatric staff can use the time‐savings for more complex tasks. However, the performance varied across different languages, disease prevalence, and question types, highlighting areas for caution.

ChatGPT achieved lower accuracy in response to the Chinese question compared to English questions, especially regarding disease definition and medication recommendation. This finding aligns with previous studies that have reported lower performance of ChatGTP in Chinese than in English, which may be attributed to the paucity of Chinese training data for the model.^22^ This result underscored the need for more diverse language training in AI models to ensure their applicability in different linguistic contexts. Clinicians and patients should exercise particular caution when referring to ChatGPT's responses to Chinese inquiries.^23^

The performance of ChatGPT was better for common diseases compared to rare diseases. This is likely due to the fact that common diseases are more frequently discussed in the literature and other sources that the model was trained on, leading to a better understanding and more accurate responses. This observation mirrored research by Schulte,^24^ which suggested a weak correlation between disease prevalence and ChatGPT's performance.^24^ In addition, the accuracy rate was slightly higher for general questions compared to specific questions, which indicated that while ChatGPT can provide useful general advice, it might struggle with more specific and complex queries, particularly those involving detailed treatment plans or medication dosages. These findings suggested that while ChatGPT can be a useful tool for providing general treatment recommendations for common pediatric diseases, its specific usage for rare diseases should be approached with caution.^25^

Although our study suggested that ChatGPT has the potential to be a useful tool in pediatric clinical practice, the error analysis revealed several areas where ChatGPT struggled, including disease definitions and recommendations for medication and surgery. Such errors could potentially lead to incorrect or even harmful advice. Furthermore, we asked ChatGPT to provide references and sources to support its responses, and while it occasionally provided some references, the titles, DOIs, and links of these references were either nonexistent or unrelated to the responses. These limitations emphasized the need for careful oversight and verification of AI‐generated recommendations in clinical practice.^26^ To maximize the benefits of chatbots without affecting the quality of care, chatbots should be viewed as a supplement to the clinical decision, not a replacement. In addition, the initial implementation of chatbots should take place in a supervised environment, and their output should be reviewed and validated by specialized clinicians prior to any clinical application. It is vital that chatbots should state their reliability in their responses and provide citations for medical statements. Finally, given the rapidly evolving nature of medical knowledge, regular updates and validations of these platforms are crucial to ensure their ongoing reliability and usefulness.

This study had several limitations. Firstly, despite the consideration of 104 pediatric diseases, the research did not encompass all conceivable clinical pediatric situations, potentially limiting the generalizability of the results to all medical scenarios. Secondly, the assessment of ChatGPT's performance occurred in a simulated environment, necessitating further investigation to evaluate its efficacy in real‐world clinical settings. Thirdly, the focus on ChatGPT's performance in Chinese and English precluded the evaluation of its performance in other languages. Additional research is required to examine its potential applications and constraints in various linguistic contexts. Finally, ChatGPT's training data extends only up to September 2021, which may pose constraints on its ability to provide the most up‐to‐date medical guidelines or recommendations. In designing our study, we were aware of this limitation and ensured that the questions posed were pertinent to guidelines and recommendations available up to that cutoff date. On a thorough review of ChatGPT's incorrect responses, we did not identify any errors that were a result of outdated medical guidelines or recommendations. Additionally, we acknowledge that newer versions of ChatGPT have capabilities to access real‐time information through specific plugins and can be fine‐tuned with locally uploaded data. However, introducing these elements into our study would have significantly increased its complexity. Such considerations, though promising, warrant exploration in future research.

In conclusion, ChatGPT demonstrates promise in providing pediatric treatment recommendations with notable accuracy; however, it is prone to errors in addressing rare diseases, specific treatment recommendations, and responses in Chinese. We should be cautiously optimistic about the potential application of advanced natural language processing applications to improve patient care.

## AUTHOR CONTRIBUTIONS

Conceptualization: Qiuhong Wei, Yanqin Wang, Zhengxiong Yao, Ximing Xu, and Tingyu Li; Formal analysis: Qiuhong Wei, Yanqin Wang, and Zhengxiong Yao; Investigation: Qiuhong Wei, Yanqin Wang, Zhengxiong Yao, Ying Cui, and Bo Wei; Methodology: Qiuhong Wei, Yanqin Wang, Zhengxiong Yao, Ying Cui, Bo Wei, and Ximing Xu; Writing the original draft: Qiuhong Wei; Writing review & editing: Yanqin Wang, Zhengxiong Yao, Ximing Xu, Bo Wei, Ying Cui, and Tingyu Li. All authors have read and agreed to the published version of the manuscript.

## CONFLICT OF INTEREST STATEMENT

The authors declare that there are no competing interests.

## ETHICS STATEMENT

Not applicable: this study did not involve human participants or use human subject data, but solely focused on the evaluation of an artificial intelligence language model. Therefore, the requirement for ethical approval was waived.

## Supporting information

Tables S1–S2

## Data Availability

Supporting data for this study are available from the corresponding author (X.M.X.) upon reasonable request.

## References

[1] Teubner T , Flath CM , Weinhardt C , van der Aalst W , Hinz O . Welcome to the era of ChatGPT et al. Bus Inform Syst Eng+. 2023;65(2):95‐101.

[2] Gilson A , Safranek CW , Huang T , et al. How does ChatGPT perform on the United States medical licensing examination? The implications of large language models for medical education and knowledge assessment. JMIR Med Educ. 2023;9:e45312. 36753318 10.2196/45312 PMC9947764

[3] Wang X , Gong Z , Wang G , et al. ChatGPT performs on the Chinese national medical licensing examination. Research Square; 2023. 10.1007/s10916-023-01961-0 37581690

[4] Korngiebel DM , Mooney SD . Considering the possibilities and pitfalls of Generative Pre‐trained Transformer 3 (GPT‐3) in healthcare delivery. NPJ Digit Med. 2021;4(1):93. 34083689 10.1038/s41746-021-00464-x PMC8175735

[5] Anonymous . Will ChatGPT transform healthcare? Nat Med. 2023;29(3):505‐506. 36918736 10.1038/s41591-023-02289-5

[6] Sallam M . ChatGPT utility in healthcare education, research, and practice: systematic review on the promising perspectives and valid concerns. Healthcare. 2023;11(6):887. 36981544 10.3390/healthcare11060887 PMC10048148

[7] Levine DM , Tuwani R , Kompa B , et al. The diagnostic and triage accuracy of the GPT‐3 artificial intelligence model. medRxiv. 2023:2023.01.30.23285067. 10.1016/S2589-7500(24)00097-9 39059888

[8] Lee TC , Staller K , Botoman V , Pathipati MP , Varma S , Kuo B . ChatGPT answers common patient questions about colonoscopy. Gastroenterology. 2023;165(2):509‐511.e7. 37150470 10.1053/j.gastro.2023.04.033

[9] Sarraju A , Bruemmer D , Van Iterson E , Cho L , Rodriguez F , Laffin L . Appropriateness of cardiovascular disease prevention recommendations obtained from a popular online chat‐based artificial intelligence model. JAMA. 2023;329(10):842‐844. 36735264 10.1001/jama.2023.1044 PMC10015303

[10] Howard A , Hope W , Gerada A . ChatGPT and antimicrobial advice: the end of the consulting infection doctor? Lancet Infect Dis. 2023;23(4):405‐406. 10.1016/S1473-3099(23)00113-5 36822213

[11] Suchman K , Garg S , Trindade AJ . ChatGPT fails the multiple‐choice American College of Gastroenterology self‐assessment test. Am J Gastroenterol. 2023. 10.14309/ajg.0000000000002320 37212584

[12] Ayers JW , Poliak A , Dredze M , et al. Comparing physician and artificial intelligence chatbot responses to patient questions posted to a public social media forum. JAMA Intern Med. 2023;183(6):589. 37115527 10.1001/jamainternmed.2023.1838 PMC10148230

[13] Haver HL , Ambinder EB , Bahl M , Oluyemi ET , Jeudy J , Yi PH . Appropriateness of breast cancer prevention and screening recommendations provided by ChatGPT. Radiology. 2023;307(4):230424. 10.1148/radiol.230424 37014239

[14] Zhu L , Mou W , Chen R . Can the ChatGPT and other large language models with internet‐connected database solve the questions and concerns of patient with prostate cancer? J Transl Med. 2023;21(1):269. 37076876 10.1186/s12967-023-04123-5 PMC10115367

[15] Cao JJ , Kwon DH , Ghaziani TT , et al. Accuracy of information provided by ChatGPT regarding liver cancer surveillance and diagnosis. AJR Am J Roentgenol. 2023;221(4):556‐559. 37222278 10.2214/AJR.23.29493

[16] Li SW , Kemp MW , Logan SJS , et al. ChatGPT outscored human candidates in a virtual objective structured clinical examination (OSCE) in obstetrics and gynecology. Am J Obstet Gynecol. 2023;229(2):172.e1‐172.e12. 10.1016/j.ajog.2023.04.020 37088277

[17] Chervenak J , Lieman H , Blanco‐Breindel M , Jindal S . The promise and peril of using a large language model to obtain clinical information: ChatGPT performs strongly as a fertility counseling tool with limitations. Fertil Steril. 2023;120(3):575‐583. 37217092 10.1016/j.fertnstert.2023.05.151

[18] Association CRDR . Announcement of the first batch of rare disease directory in China. Accessed October 11, 2023. https://www.gov.cn/

[19] Mihalache A , Popovic MM , Muni RH . Performance of an artificial intelligence chatbot in ophthalmic knowledge assessment. JAMA Ophthalmol. 2023;141(6):589. 37103928 10.1001/jamaophthalmol.2023.1144 PMC10141269

[20] Duong D , Solomon BD . Analysis of large‐language model versus human performance for genetics questions. Eur J Hum Genet. 2023. 10.1038/s41431-023-01396-8 PMC10999420 37246194

[21] Hirosawa T , Harada Y , Yokose M , Sakamoto T , Kawamura R , Shimizu T . Diagnostic accuracy of differential‐diagnosis lists generated by generative pretrained transformer 3 chatbot for clinical vignettes with common chief complaints: a pilot study. Int J Environ Res Publ Health. 2023;20(4):3378. 10.3390/ijerph20043378 PMC9967747 36834073

[22] Wang YM , Shen HW , Chen TJ . Performance of ChatGPT on the pharmacist licensing examination in Taiwan. J Chin Med Assoc. 2023;86(7):653‐658. 37227901 10.1097/JCMA.0000000000000942 PMC12755457

[23] Shen Y , Heacock L , Elias J , et al. ChatGPT and other large language models are double‐edged swords. Radiology. 2023;307(2):230163. 10.1148/radiol.230163 36700838

[24] Schulte B . Capacity of ChatGPT to identify guideline‐based treatments for advanced solid tumors. Cureus. 2023;15(4):e37938. 37220429 10.7759/cureus.37938 PMC10200252

[25] Baumgartner C . The potential impact of ChatGPT in clinical and translational medicine. Clin Transl Med. 2023;13(3):e1206. 36854881 10.1002/ctm2.1206 PMC9974599

[26] Li J , Dada A , Kleesiek J , Egger J . ChatGPT in healthcare: a taxonomy and systematic review. medRxiv. 2023. 10.1101/2023.03.30.23287899 38262126

