# Supplementary material for: Evaluation of ChatGPT's performance in providing treatment recommendations for pediatric diseases
Source: Pediatr Discov. 2023 Nov 20;1(3):e42. doi: 10.1002/pdi3.42 (PMC12118210; doi:10.1002/pdi3.42)
Supplement: Supplementary file 1 — Tables S1–S2 [file PDI3-1-e42-s001.docx]

Supplementary Material

| Supplementary Table 1. List of Questions Posed to ChatGPT | | | | |
| --- | --- | --- | --- | --- |
| Serial No. | General Question in English | Specific Question in English | General Question in Chinese | Specific Question in Chinese |
| 1 | A child diagnosed with central precocious puberty, how to treat? | A 7-year-old child, 20 kg, diagnosed with central precocious puberty, which drug is preferred for treatment and what is the specific dosage? | 一名儿童诊断为中枢性性早熟，请问如何治疗 | 一名7岁儿童，20kg，诊断为中枢性性早熟，首选哪种药物进行治疗，具体的用法用量是？ |
| 2 | A child diagnosed with Graves' disease, how to treat? | A 7-year-old child, 21 kg, diagnosed with Graves' disease, which drug is preferred for treatment and what is the specific dosage? | 一名儿童诊断为Graves病，请问如何治疗 | 一名7岁儿童，20kg，诊断为Graves病，首选哪种药物进行治疗，具体的用法用量是？ |
| 3 | A child diagnosed with congenital hypothyroidism, how to treat? | A 3-year-old child, 14 kg, diagnosed with congenital hypothyroidism, which drug is preferred for treatment and what is the specific dosage? | 一名儿童诊断为先天性甲状腺功能减退症，请问如何治疗？ | 一名3岁儿童，14kg，诊断为先天性甲状腺功能减退症，首选哪种药物进行治疗，具体的用法用量是？ |
| 4 | A child diagnosed with congenital adrenal hyperplasia, how to treat? | A 3-year-old child, 14 kg, diagnosed with congenital adrenal hyperplasia, which drug is preferred for treatment and what is the specific dosage? | 一名儿童诊断为先天性肾上腺皮质增生症，请问如何治疗？ | 一名3岁儿童，14kg，诊断为先天性肾上腺皮质增生症，首选哪种药物进行治疗，具体的用法用量是？ |
| 5 | A child diagnosed with Tourette syndrome, how to treat? | A 7-year-old child, 20 kg, diagnosed with Tourette's syndrome, which drug is preferred for treatment and what is the specific dosage? | 一名儿童诊断为抽动秽语综合征，请问如何治疗 | 一名7岁儿童，20kg，诊断为抽动秽语综合征，首选哪种药物进行治疗，具体的用法用量是？ |
| 6 | A child diagnosed with Myasthenia gravis ocular muscle type, how to treat? | A 7-year-old child, 20 kg, diagnosed with Myasthenia gravis ocular muscle type, which drug is preferred for treatment and what is the specific dosage? | 一名儿童诊断为重症肌无力眼肌型，请问如何治疗 | 一名7岁儿童，20kg，诊断为重症肌无力眼肌型，首选哪种药物进行治疗，具体的用法用量是？ |
| 7 | A child diagnosed with Febrile seizures, how to treat? | A 3-year-old child, 14 kg, diagnosed with Febrile seizures, which drug is preferred for treatment and what is the specific dosage? | 一名儿童诊断为热性惊厥单纯型，请问如何治疗 | 一名3岁儿童，14kg，诊断为热性惊厥单纯型，首选哪种药物进行治疗，具体的用法用量是？ |
| 8 | A infant diagnosed with infantile spasms, how to treat? | A 8-month-old infant, 8 kg, diagnosed with infantile spasms, which drug is preferred for treatment and what is the specific dosage? | 一名儿童诊断为婴儿痉挛症，请问如何治疗？ | 一名11月婴儿，8kg，诊断为婴儿痉挛症，首选哪种药物进行治疗，具体的用法用量？ |
| 9 | A child diagnosed with nephrotic syndrome, how to treat? | A 7-year-old child, 20 kg, diagnosed with steroid sensitive nephrotic syndrome, which drug is preferred for treatment and what is the specific dosage? | 一名儿童诊断为肾病综合征，请问如何治疗 | 一名7岁儿童，20kg，诊断为激素敏感型肾病综合征，首选哪种药物进行治疗，具体的用法用量是？ |
| 10 | A child diagnosed with Henoch-Schönlein purpura nephritis, how to treat? | A 7-year-old child, 20 kg, diagnosed with Henoch-Schönlein purpura nephritis, which drug is preferred for treatment and what is the specific dosage? | 一名儿童诊断为过敏性紫癜性肾炎，请问如何治疗 | 一名7岁儿童，20kg，诊断为过敏性紫癜性肾炎，首选哪种药物进行治疗，具体的用法用量是？ |
| 11 | A child diagnosed with urinary tract infections, how to treat? | A 3-year-old child, 14 kg, diagnosed with urinary tract infections, which drug is preferred for treatment and what is the specific dosage? | 一名儿童诊断为泌尿道感染，请问如何治疗? | 一名3岁儿童，14kg，诊断为泌尿道感染，首选哪种药物进行治疗，具体的用法用量是？ |
| 12 | A child diagnosed with Alport syndrome, how to treat? | A 3-year-old child, 14 kg, diagnosed with Alport syndrome, what is the preferred treatment? | 一名儿童诊断为Alport 综合征，请问如何治疗 | 一名3岁儿童，14kg，诊断为Alport 综合征，首选什么治疗？ |
| 13 | A newborn diagnosed with neonatal omphalitis, how to treat? | A 20-day-old newborn, 8 kg, diagnosed with neonatal omphalitis, which drug is preferred for treatment and what is the specific dosage? | 一名新生儿诊断为新生儿脐炎，请问如何治疗？ | 一名20天新生儿，4kg，诊断为新生儿脐炎，首选哪种药物进行治疗，具体的用法用量？ |
| 14 | A newborn diagnosed with neonate pneumonia, how to treat? | A 20-day-old newborn, 8 kg, diagnosed with neonate pneumonia, which drug is preferred for treatment and what is the specific dosage? | 一名新生儿诊断为新生儿肺炎，请问如何治疗？ | 一名20天新生儿，4kg，诊断为新生儿肺炎，首选哪种药物进行治疗，具体的用法用量？ |
| 15 | A newborn diagnosed with neonatal hyperbilirubinemia, how to treat? | A 20-day-old newborn, 8 kg, diagnosed with neonatal hyperbilirubinemia, which drug is preferred for treatment and what is the specific dosage? | 一名新生儿诊断为新生儿高胆红素血症，请问如何治疗？ | 一名20天新生儿，4kg，诊断为新生儿高胆红素血症，首选哪种药物进行治疗，具体的用法用量？ |
| 16 | A newborn diagnosed with Congenital Myasthenic Syndrome., how to treat? | A 20-day-old newborn, 8 kg, diagnosed with Congenital Myasthenic Syndrome, which drug is preferred for treatment and what is the specific dosage? | 一名新生儿诊断为先天性肌无力综合征，请问如何治疗？ | 一名20天新生儿，4kg，诊断为先天性肌无力综合征，首选哪种药物进行治疗，具体的用法用量？ |
| 17 | A child diagnosed with premature ventricualr contraction, how to treat? | A 7-year-old child, 20 kg, diagnosed with premature ventricualr contraction, which drug is preferred for treatment and what is the specific dosage? | 一名儿童诊断为室性早博，请问如何治疗? | 一名7岁儿童，20kg，诊断为室性早博，首选哪种药物进行治疗，具体的用法用量是？ |
| 18 | A child diagnosed with atrial septal defect, how to treat? | A 2-year-old child, 12 kg, diagnosed with atrial septal defect, what is the preferred treatment and what is the specific treatment? | 一名儿童诊断为房间隔缺损，请问如何治疗 | 一名2岁儿童，12kg，诊断为房间隔缺损，首选什么治疗，具体治疗方法是什么？ |
| 19 | A child diagnosed with patent foramen ovale, how to treat? | A 4-year-old child, 16 kg, diagnosed with patent foramen ovale, what is the preferred treatment and what is the specific treatment? | 一名儿童诊断为卵圆孔未闭，请问如何治疗？ | 一名4岁儿童，16kg，诊断为卵圆孔未闭，首选什么治疗，具体治疗方法是什么？ |
| 20 | A child diagnosed with Brugada syndrome, how to treat? | A 12-year-old child, 40 kg, diagnosed with Bruguada syndrome, what is the preferred treatment and what is the specific treatment? | 一名儿童诊断为布鲁加达综合征，请问如何治疗？ | 一名12岁儿童，40kg，诊断为布鲁加达综合征，首选什么治疗，具体治疗方法是什么？ |
| 21 | A child diagnosed with Acute Lymphoblastic Leukemia, how to treat? | A 7-year-old child, 20 kg, diagnosed with Acute Lymphoblastic Leukemia, which drug is preferred for treatment and what is the specific dosage? | 一名儿童诊断为急性淋巴细胞白血病，请问如何治疗 | 一名7岁儿童，20kg，诊断为急性淋巴细胞白血病，首选哪种药物进行治疗，具体的用法用量是？ |
| 22 | A child diagnosed with idiopathic thrombocytopenic purpura, how to treat? | A 7-year-old child, 20 kg, diagnosed with idiopathic thrombocytopenic purpura, which drug is preferred for treatment and what is the specific dosage? | 一名儿童诊断为原发性免疫性血小板减少症，请问如何治疗 | 一名7岁儿童，20kg，诊断为原发性免疫性血小板减少症，首选哪种药物进行治疗，具体的用法用量是？ |
| 23 | A child diagnosed with iron deficiency anemia, how to treat? | A 7-year-old child, 20 kg, diagnosed with iron deficiency anemia, which drug is preferred for treatment and what is the specific dosage? | 一名儿童诊断为缺铁性贫血，请问如何治疗 | 一名7岁儿童，20kg，诊断为缺铁性贫血，首选哪种药物进行治疗，具体的用法用量是？ |
| 24 | A child diagnosed with Fanconi anemia, how to treat? | A 7-year-old child, 20 kg, diagnosed with Fanconi anemia, which drug is preferred for treatment and what is the specific dosage? | 一名儿童诊断为范可尼贫血，请问如何治疗 | 一名7岁儿童，20kg，诊断为范可尼贫血，首选哪种药物进行治疗，具体的用法用量是？ |
| 25 | A child diagnosed with Subarachnoid Hemorrhage, how to treat? | A 7-year-old child, 20 kg, diagnosed with Subarachnoid Hemorrhage, which surgical procedure is preferred for treatment? | 一名儿童诊断为蛛网膜下腔出血，请问如何治疗 | 一名7岁儿童，20kg，诊断为蛛网膜下腔出血，首选哪种手术进行治疗？ |
| 26 | A child diagnosed with Arachnoid cysts, how to treat? | A 7-year-old child, 20 kg, diagnosed with Arachnoid cysts, which surgical procedure is preferred for treatment? | 一名儿童诊断为蛛网膜囊肿，请问如何治疗 | 一名7岁儿童，20kg，诊断为蛛网膜囊肿，首选哪种手术进行治疗？ |
| 27 | A child diagnosed with Hydrocephalus, how to treat? | A 1-year-old child, 8 kg, diagnosed with Hydrocephalus, which surgical procedure is preferred for treatment? | 一名儿童诊断为脑积水，请问如何治疗 | 一名1岁儿童，8kg，诊断为脑积水，首选哪种手术进行治疗？ |
| 28 | A child diagnosed with Neurofibromatosis Type 1, how to treat? | A 7-year-old child, 20 kg, diagnosed with Neurofibromatosis Type 1, which surgical procedure is preferred for treatment? | 一名儿童诊断为Ⅰ型神经纤维瘤病，请问如何治疗 | 一名7岁儿童，20kg，诊断为Ⅰ型神经纤维瘤病，首选哪种手术进行治疗？ |
| 29 | A child diagnosed with perianal abscess, how to treat? | A 1-year-old child, 8 kg, diagnosed with perianal abscess, which surgical procedure is preferred for treatment? | 一名儿童诊断为肛周脓肿，请问如何治疗 | 一名1岁儿童，8kg，诊断为肛周脓肿，首选哪种手术进行治疗？ |
| 30 | A child diagnosed with anal fissure, how to treat? | A 1-year-old child, 8 kg, diagnosed with anal fissure, which surgical procedure is preferred for treatment? | 一名儿童诊断为肛裂，请问如何治疗 | 一名1岁儿童，8kg，诊断为肛裂，首选哪种手术进行治疗？ |
| 31 | A child diagnosed with Intussusception, how to treat? | A 1-year-old child, 8 kg, diagnosed with Intussusception, which surgical procedure is preferred for treatment? | 一名儿童诊断为肠套叠，请问如何治疗 | 一名1岁幼儿，8kg，诊断为肠套叠，首选哪种手术进行治疗？ |
| 32 | A child diagnosed with Gastrointestinal stromal tumor, how to treat? | A 10-year-old child, 25 kg, diagnosed with Gastrointestinal stromal tumor, which surgical procedure is preferred for treatment? | 一名儿童诊断为胃肠间质瘤，请问如何治疗 | 一名10岁儿童，25kg，诊断为胃肠间质瘤，首选哪种手术进行治疗？ |
| 33 | A child diagnosed with pectus excavatum, how to treat? | A 9-year-old child, 30 kg, diagnosed with pectus excavatum, which surgery is preferred for treatment? | 一名儿童诊断为漏斗胸，请问如何治疗 | 一名9岁儿童，30kg，诊断为漏斗胸，首选哪种手术进行治疗？ |
| 34 | A child diagnosed with tuberculous pleural effusion, how to treat? | A 7-year-old child, 20 kg, diagnosed with tuberculous pleural effusion, which is the preferred surgical treatment and what is the specific treatment plan? | 一名儿童诊断为结核性胸腔积液，请问如何治疗? | 一名7岁儿童，20kg，诊断为结核性胸腔积液，首选哪种外科治疗，具体的治疗方案是什么？ |
| 35 | A child diagnosed with tetralogy of Fallot, how to treat? | A 3-month-old child, 5 kg, diagnosed with tetralogy of Fallot, which is the preferred surgical treatment? | 一名儿童诊断为法洛四联症，请问如何治疗 | 一名3月龄儿童，5kg，诊断为法洛四联症，首选哪种手术治疗？ |
| 36 | A child diagnosed with Marfan syndrome, how to treat? | A 7-year-old child, 20 kg, diagnosed with Marfan syndrome, what are the surgical treatments? | 一名儿童诊断为马方综合征，请问如何治疗 | 一名7岁儿童，20kg，诊断为马方综合征，外科治疗方法有哪些？ |
| 37 | A child diagnosed with neuroblastoma, how to treat? | A 3-year-old child, 14 kg, diagnosed with neuroblastoma, what is the surgical treatment? | 一名儿童诊断为神经母细胞瘤，请问如何治疗 | 一名3岁儿童，14kg，诊断为神经母细胞瘤，如何进行外科治疗？ |
| 38 | A child diagnosed with lipoma, how to treat? | A 3-year-old child, 14 kg, diagnosed with superficial lipoma of the shoulder, what is the surgical treatment? | 一名儿童诊断为脂肪瘤，请问如何治疗 | 一名3岁儿童，14kg，诊断为肩部浅表脂肪瘤，如何进行外科治疗？ |
| 39 | A child diagnosed with melanoma, how to treat? | A 7-year-old child, 20 kg, diagnosed with melanoma, what is the surgical treatment? | 一名儿童诊断为黑色素瘤，请问如何治疗 | 一名7岁儿童，20kg，诊断为黑色素瘤，首选哪种外科治疗？ |
| 40 | A child diagnosed with nephroblastoma, how to treat? | A 3-year-old child, 14 kg, diagnosed with nephroblastoma, what is the surgical treatment? | 一名儿童诊断为肾母细胞瘤，请问如何治疗 | 一名3岁儿童，14kg，诊断为肾母细胞瘤，首选哪种外科治疗？ |
| 41 | A child diagnosed with Vitamin D deficiency, how to treat? | A 3-year-old child, 14 kg, diagnosed with Vitamin D deficiency, which drug is preferred for treatment and what is the specific dosage? | 一名儿童诊断为维生素D缺乏症，请问如何治疗 | 一名3岁儿童，14kg，诊断为维生素D缺乏症，首选哪种药物进行治疗，具体的用法用量是？ |
| 42 | A child diagnosed with mild malnutrition, how to treat? | A 3-year-old boy, 12 kg, diagnosed with mild malnutrition, what is preferred treatment? | 一名儿童诊断为轻度营养不良，请问如何治疗 | 一名3岁儿童，12kg，诊断为轻度营养不良，首选什么治疗，具体的治疗方法是？ |
| 43 | A child diagnosed with food allergy, how to treat? | A 1-year-old child, 8 kg, diagnosed with food allergy, what is preferred treatment? | 一名儿童诊断为食物蛋白过敏，请问如何治疗 | 一名1岁儿童，8kg，诊断为食物蛋白过敏，首选什么治疗，具体的治疗方式是什么？ |
| 44 | A child diagnosed with X-linked hypophosphatemic rickets, how to treat? | A 7-year-old child, 20 kg, diagnosed with X-linked hypophosphatemic rickets, which drug is preferred for treatment and what is the specific dosage? | 一名儿童诊断为X连锁低磷性佝偻病，请问如何治疗 | 一名7岁儿童，20kg，诊断为X连锁低磷性佝偻病，首选哪种药物进行治疗，具体的用法用量是？ |
| 45 | A child diagnosed with ADHD, how to treat? | A 7-year-old child, 20 kg, diagnosed with ADHD, which drug is preferred for treatment and what is the specific dosage? | 一名儿童诊断为注意缺陷与多动障碍，请问如何治疗？ | 一名7岁儿童，20kg，诊断为注意缺陷与多动障碍，首选哪种药物进行治疗，具体的用法用量是？ |
| 46 | A child diagnosed with Global Developmental Delay, how to treat? | A 3-year-old child, 14 kg, diagnosed with global developmental delay, what is the preferred treatment? | 一名儿童诊断为全面发育迟缓，请问如何治疗？ | 一名3岁儿童，14kg，诊断为全面发育迟缓，首选什么治疗？ |
| 47 | A child diagnosed with ASD, how to treat? | A 3-year-old child, 14 kg, diagnosed with ASD, what is the preferred treatment? | 一名儿童诊断为孤独症谱系障碍，请问如何治疗？ | 一名3岁儿童，14kg，诊断为孤独症谱系障碍，首选什么治疗？ |
| 48 | A child diagnosed with Rett syndrome, how to treat? | A 3-year-old child, 14 kg, diagnosed with Rett syndrome, what is the preferred treatment? | 一名儿童诊断为Rett综合征，请问如何治疗？ | 一名3岁儿童，14kg，诊断为Rett综合征，首选什么治疗？ |
| 49 | A child diagnosed with Refractive error, how to treat? | A 7-year-old child, 20 kg, diagnosed with Refractive error, How to treat and what is the specific method for the preferred treatment? | 一名儿童诊断为屈光不正，请问如何治疗 | 一名7岁儿童，20kg，诊断为屈光不正，首选哪种治疗方法，具体的方法是？ |
| 50 | A child diagnosed with Allergic Conjunctivitis, how to treat? | A 7-year-old child, 20 kg, diagnosed with Allergic Conjunctivitis, which drug is preferred for treatment and what is the specific dosage? | 一名儿童诊断为过敏性结膜炎，请问如何治疗 | 一名7岁儿童，20kg，诊断为过敏性结膜炎，首选哪种药物进行治疗，具体的用法用量是？ |
| 51 | A child diagnosed with Dry eyes, how to treat? | A 7-year-old child, 20 kg, diagnosed with Dry eyes, which drug is preferred for treatment and what is the specific dosage? | 一名儿童诊断为干眼症，请问如何治疗 | 一名7岁儿童，20kg，诊断为干眼症，首选哪种药物进行治疗，具体的用法用量是？ |
| 52 | A child diagnosed with Leber's hereditary optic neuropathy, how to treat? | A 7-year-old child, 20 kg, diagnosed with Leber's hereditary optic neuropathy, which drug is preferred for treatment and what is the specific dosage? | 一名儿童诊断为Leber 遗传性视神经病，请问如何治疗 | 一名7岁儿童，20kg，诊断为Leber 遗传性视神经病，首选哪种药物进行治疗，具体的用法用量是？ |
| 53 | A child diagnosed with Kawasaki disease, how to treat | A 4-year-old child, 14kg, was diagnosed with Kawasaki disease,which drug is preferred for treatment and what is the specific dosage? | 一名儿童诊断为川崎病，请问如何治疗 | 一名4岁儿童，14kg，诊断为川崎病，首选哪种药物进行治疗，具体的用法用量是？ |
| 54 | A child diagnosed with systemic lupus erythematosus, how to treat it | A 10-year-old child, 25kg, was diagnosed with systemic lupus erythematosus,which drug is preferred for treatment and what is the specific dosage? | 一名儿童诊断为系统性红斑狼疮，请问如何治疗 | 一名10岁儿童，25kg，诊断为系统性红斑狼疮，首选哪种药物进行治疗，具体的用法用量是？ |
| 55 | A child diagnosed with the first onset of juvenile idiopathic arthritis oligoarthritis, how to treat it | A 5-year-old child, 18kg, was diagnosed as the first-onset juvenile idiopathic arthritis oligoarthritis,which drug is preferred for treatment and what is the specific dosage? | 一名儿童诊断为初发的幼年特发性关节炎少关节型，请问如何治疗 | 一名5岁儿童，18kg，诊断为初发的幼年特发性关节炎少关节型，首选哪种药物进行治疗，具体的用法用量是？ |
| 56 | How is a child diagnosed with X-linked agammaglobulinemia treated? | A 5-year-old child, 18kg, diagnosed with X-linked agammaglobulinemia, which drug is preferred for treatment and what is the specific dosage? | 一名儿童诊断为X-连锁无丙种球蛋白血症，请问如何治疗 | 一名5岁儿童，18kg，诊断为X-连锁无丙种球蛋白血症，首选哪种药物进行治疗，具体的用法用量是？ |
| 57 | A child is diagnosed with hand, foot and mouth disease, how to treat it | A 5-year-old child, 18kg, was diagnosed with hand, foot and mouth, What is the preferred treatment and specific treatment plan? | 一名儿童诊断为手足口病，请问如何治疗 | 一名3岁儿童，15kg，诊断为手足口病，首选何种治疗，具体的治疗方案是什么？ |
| 58 | A child is diagnosed with chickenpox, how to treat it | A 5-year-old child, 18kg, was diagnosed with chickenpox, which drug is preferred for treatment and what is the specific dosage? | 一名儿童诊断为水痘，请问如何治疗 | 一名5岁儿童，18kg，诊断为水痘，首选哪种药物进行治疗，具体的用法用量是？ |
| 59 | A child is diagnosed with pertussis, how is it treated | A 2-month-old child, 5kg, was diagnosed with pertussis, which drug is preferred for treatment and what is the specific dosage? | 一名儿童诊断为百日咳，请问如何治疗 | 一名2月龄儿童，5kg，诊断为百日咳，首选哪种药物进行治疗，具体的用法用量是？ |
| 60 | A child was diagnosed with hepatolenticular degeneration, how to treat it | A 10-year-old child, 25kg, was diagnosed with hepatolenticular degeneration, which drug is preferred for treatment and what is the specific dosage? | 一名儿童诊断为肝豆状核变性，请问如何治疗 | 一名10岁儿童，25kg，诊断为肝豆状核变性，首选哪种药物进行治疗，具体的用法用量是？ |
| 61 | A child was diagnosed with bacterial dysentery. How can he be treated? | A 5-year-old child, 18kg, was diagnosed with bacterial dysentery, which drug is preferred for treatment and what is the specific dosage? | 一名儿童诊断为细菌性痢疾，请问如何治疗？ | 一名5岁儿童，18kg，诊断为细菌性痢疾，首选哪种药物进行治疗，具体的用法用量是？ |
| 62 | A child is diagnosed with constipation, how to treat it | A 5-year-old child, 18kg, was diagnosed with constipation, which drug is preferred for treatment and what is the specific dosage? | 一名儿童诊断为便秘，请问如何治疗 | 一名5岁儿童，18kg，诊断为便秘，首选哪种药物进行治疗，具体的用法用量是？ |
| 63 | A child is diagnosed with indigestion, how to treat it | A 5-year-old child, 18kg, was diagnosed with indigestion, which drug is preferred for treatment and what is the specific dosage? | 一名儿童诊断为消化不良，请问如何治疗 | 一名5岁儿童，18kg，诊断为消化不良，首选哪种药物进行治疗，具体的用法用量是？ |
| 64 | A child diagnosed with Gaucher disease, how to treat it | A 2-year-old child, 12kg, was diagnosed with Gaucher disease, which drug is preferred for treatment and what is the specific dosage? | 一名儿童诊断为戈谢病，请问如何治疗 | 一名2岁儿童，12kg，诊断为戈谢病，首选哪种药物进行治疗，具体的用法用量是？ |
| 65 | A child was diagnosed with acute upper respiratory tract infection caused by influenza A virus infection, how to treat it | A 5-year-old child, 18kg, was diagnosed with acute upper respiratory tract infection caused by influenza A virus infection, which drug is preferred for treatment and what is the specific dosage? | 一名儿童诊断为甲型流感病毒感染导致的急性上呼吸道感染，请问如何治疗 | 一名5岁儿童，18kg，诊断为甲型流感病毒感染导致的急性上呼吸道感染，首选哪种药物进行治疗，具体的用法用量是？ |
| 66 | A child is diagnosed with acute bronchitis, how to treat it | A 5-year-old child, 18kg, was diagnosed with acute bronchitis, which drug is preferred for treatment and what is the specific dosage? | 一名儿童诊断为急性支气管炎，请问如何治疗 | 一名5岁儿童，18kg，诊断为急性支气管炎，首选哪种药物进行治疗，具体的用法用量是？ |
| 67 | A child is diagnosed with mycoplasma pneumonia, how to treat it | A 5-year-old child, 18kg, was diagnosed with mycoplasma pneumonia, which drug is preferred for treatment and what is the specific dosage? | 一名儿童诊断为支原体肺炎，请问如何治疗 | 一名5岁儿童，18kg，诊断为支原体肺炎，首选哪种药物进行治疗，具体的用法用量是？ |
| 68 | A child diagnosed with alveolar proteinosis, how to treat it | A 10-year-old child, 25kg, was diagnosed with alveolar proteinosis, how to perform whole lung lavage | 一名儿童诊断为肺泡蛋白沉积症，请问如何治疗 | 一名5岁儿童，18kg，诊断为肺泡蛋白沉积症，首选哪种药物进行治疗，具体的用法用量是？ |
| 69 | A child diagnosed with motor delay, how to treat it | A 2-year-old child, 12kg, is diagnosed with motor developmental delay,which treatment is the first choice, and what is the specific treatment plan? | 一名儿童诊断为运动发育迟缓，请问如何治疗 | 一名2岁儿童，12kg，诊断为运动发育迟缓，首选何种治疗，具体的治疗方案是？ |
| 70 | A child is diagnosed with cerebral palsy, how to treat it | A 10-year-old child, 25kg, diagnosed with cerebral palsy, which treatment is the first choice, and what is the specific treatment plan? | 一名儿童诊断为脑瘫，请问如何治疗 | 一名2岁儿童，12kg，诊断脑瘫，首选什么治疗，具体的治疗方案？ |
| 71 | A child diagnosed with sequelae of Japanese encephalitis, how to treat it? | A 10-year-old child, 28kg, diagnosed Japanese encephalitis sequelae, what is the first choice of treatment, what is the specific treatment plan? | 一名儿童诊断为流行性乙型脑炎后遗症，请问如何治疗 | 一名10岁儿童，28kg，诊断流行性乙型脑炎后遗症，请问首选什么治疗，具体的治疗方案是什么？ |
| 72 | How is a child diagnosed with X-linked adrenoleukodystrophy treated? | A 10-year-old child, 25kg, diagnosed with X-linked adrenoleukodystrophy, which treatment is the first choice, and what is the specific treatment plan? | 一名儿童诊断为X-连锁肾上腺脑白质营养不良，请问如何治疗 | 一名10岁儿童，28kg，诊断X-连锁肾上腺脑白质营养不良，首选什么治疗，具体的治疗方案？ |
| 73 | A child is diagnosed with acute cholangitis, how to treat it | A 10-year-old child, 25kg, diagnosed with acute cholangitis, which treatment is the first choice, and what is the specific treatment plan? | 一名儿童诊断为急性胆管炎，请问如何治疗 | 一名10岁儿童，28kg，诊断急性胆管炎，首选什么治疗，具体的治疗方案如何？ |
| 74 | A child is diagnosed with acute cholecystitis, how to treat it | A 10-year-old child, 25kg, was diagnosed with acute cholecystitis, which treatment is the first choice, and what is the specific treatment plan? | 一名儿童诊断为急性胆囊炎，请问如何治疗 | 一名10岁儿童，28kg，诊断急性胆囊炎，首选什么治疗，具体的治疗方案如何？ |
| 75 | A child is diagnosed with bile duct stones, how to treat them | A 10-year-old child, 25kg, was diagnosed with bile duct stones,which treatment is the first choice, and what is the specific treatment plan? | 一名儿童诊断为胆管结石，请问如何治疗 | 一名10岁儿童，28kg，诊断胆管结石，首选什么治疗，具体的治疗方案如何？ |
| 76 | A child diagnosed with congenital bile acid synthesis disorder, how to treat it | A 1-year-old child, 11 kg, diagnosed with congenital bile acid synthesis disorder. What surgical treatment is preferred and what is the specific treatment plan? | 一名儿童诊断为先天性胆汁酸合成障碍，请问如何治疗 | 一名1岁儿童，11kg，诊断为先天性胆汁酸合成障碍，首选什么外科治疗，具体的治疗方案如何？ |
| 77 | A child is diagnosed with a forearm fracture, how is it treated | A 10-year-old child, 25kg, was diagnosed with a forearm fracture, which treatment is the first choice, and what is the specific treatment plan? | 一名儿童诊断为前臂骨折，请问如何治疗 | 一名10岁儿童，28kg，诊断为前臂骨折，首选什么治疗，具体的治疗方案如何？ |
| 78 | A child diagnosed with scoliosis, how to treat it | A 10-year-old child, 25kg, diagnosed with scoliosis, which treatment is the first choice, and what is the specific treatment plan? | 一名儿童诊断为脊柱侧弯，请问如何治疗 | 一名10岁儿童，28kg，诊断脊柱侧弯，首选什么治疗，具体的治疗方案如何？ |
| 79 | A child diagnosed with congenital dysplasia of the hip, how is it treated | A 1-year-old child, 11kg, was diagnosed with congenital dysplasia of the hip joint.which treatment is the first choice, and what is the specific treatment plan? | 一名儿童诊断为先天性髋关节发育不良，请问如何治疗 | 一名1岁儿童，11kg，诊断先天性髋关节发育不良，首选什么治疗，具体的治疗方案如何？ |
| 80 | A child diagnosed with congenital scoliosis, how to treat it | A 10-year-old child, 25kg, diagnosed with congenital scoliosis, which treatment is the first choice, and what is the specific treatment plan? | 一名儿童诊断为先天性脊柱侧凸，请问如何治疗 | 一名10岁儿童，28kg，诊断先天性脊柱侧凸，首选什么治疗，具体的治疗方案如何？ |
| 81 | A child is diagnosed with redundant prepuce, how to treat it | A 10-year-old child, 25kg, was diagnosed with excessive prepuce,which treatment is the first choice, and what is the specific treatment plan? | 一名儿童诊断为包皮过长，请问如何治疗 | 一名10岁儿童，28kg，诊断包皮过长，首选什么治疗，具体的治疗方案如何？ |
| 82 | A child is diagnosed with Hydrocele of tunica vaginalis, how to treat it | A 10 year old child, 25kg, diagnosed with Hydrocele of tunica vaginalis, which treatment is the first choice, and what is the specific treatment plan? | 一名儿童诊断为鞘膜积液，请问如何治疗 | 一名10岁儿童，28kg，诊断鞘膜积液，首选什么治疗，具体的治疗方案如何？ |
| 83 | A child has been diagnosed with an inguinal hernia, how to treat it | A 10-year-old child, 28kg, was diagnosed with an indirect inguinal hernia,which treatment is the first choice, and what is the specific treatment plan? | 一名儿童诊断为腹股沟斜疝，请问如何治疗 | 一名10岁儿童，28kg，诊断为腹股沟斜疝，首选什么治疗，具体的治疗方案如何？ |
| 84 | A Child Diagnosed with Prader-Willi Syndrome, How to Treat It | A 5-year-old male child, 18kg, was diagnosed with Prader-Willi syndrome, which treatment is the first choice, and what is the specific treatment plan? | 一名儿童诊断为普拉德-威利综合征，请问如何治疗 | 一名5岁男性儿童，25kg，诊断为普拉德-威利综合征，首选什么治疗，具体的治疗方案如何？ |
| 85 | A newborn was diagnosed as Hirschsprung, how to treat it | A 10-day-old newborn, weighing 3.8kg, was diagnosed with Hirschsprung’s disease.which treatment is the first choice, and what is the specific treatment plan? | 一名新生儿诊断为先天性巨结肠，请问如何治疗 | 一名10日龄新生儿，3.8kg，诊断为先天性巨结肠，首选什么治疗，具体的治疗方案如何？ |
| 86 | A newborn was diagnosed as neonatal necrotizing enterocolitis Bell stage III, how to treat it | A 10-day-old newborn, weighing 3.8kg, was diagnosed with neonatal necrotizing enterocolitis Bell stage III,which treatment is the first choice, and what is the specific treatment plan? | 一名新生儿诊断为新生儿坏死性小肠结肠炎Bell分期Ⅲ期，请问如何治疗 | 一名10日龄新生儿，3.8kg，诊断为新生儿坏死性小肠结肠炎Bell分期Ⅲ期，首选什么治疗，具体的治疗方案如何？ |
| 87 | A newborn is diagnosed with umbilical hernia, how to treat it | A 10-day-old newborn, 3.8kg, was diagnosed with umbilical hernia,which treatment is the first choice, and what is the specific treatment plan? | 一名新生儿诊断为脐疝，请问如何治疗 | 一名10日龄新生儿，3.8kg，诊断为脐疝，首选什么治疗，具体的治疗方案如何？ |
| 88 | A newborn was diagnosed with congenital coronary aneurysm, how to treat it | A 10-day-old newborn, weighing 3.8kg, was diagnosed with a congenital coronary aneurysm.which treatment is the first choice, and what is the specific treatment plan? | 一名新生儿诊断为先天性冠状动脉瘤，请问如何治疗 | 一名10日龄新生儿，3.8kg，诊断为先天性冠状动脉瘤，首选什么治疗，具体的治疗方案如何？ |
| 89 | A child was diagnosed with third-degree burns on both thighs, how to treat them | A 10-year-old child, weighing 28kg, was diagnosed with third-degree burns on both thighs.which treatment is the first choice, and what is the specific treatment plan? | 一名儿童诊断为双侧大腿Ⅲ度烫伤，请问如何治疗 | 一名10岁儿童，28kg，诊断双侧大腿Ⅲ度烫伤，首选什么治疗，具体的治疗方案如何？ |
| 90 | A child is diagnosed with cleft lip and palate, how to treat it | A 1-year-old child, 11kg, diagnosed with cleft lip and palate,which treatment is the first choice, and what is the specific treatment plan? | 一名儿童诊断为唇腭裂，请问如何治疗 | 一名1岁儿童，11kg，诊断唇腭裂，首选什么治疗，具体的治疗方案如何？ |
| 91 | A child was diagnosed with facial scar hyperplasia, how to treat it | A 10-year-old child, 28kg, was diagnosed with facial scar hyperplasia, which treatment is the first choice, and what is the specific treatment plan? | 一名儿童诊断为面部瘢痕增生，请问如何治疗 | 一名10岁儿童，28kg，诊断面部瘢痕增生，首选什么治疗，具体的治疗方案如何？ |
| 92 | A child diagnosed with a congenital melanocytic nevus, how to treat | A 5-year-old child, 18kg, was diagnosed with congenital melanocytic nevus,which treatment is the first choice, and what is the specific treatment plan? | 一名儿童诊断为先天性黑色素细胞痣，请问如何治疗 | 一名5岁儿童，18kg，诊断为先天性黑色素细胞痣，首选什么治疗，具体的治疗方案如何？ |
| 93 | A child diagnosed with allergic rhinitis, how to treat it | A 10-year-old child, 28kg, was diagnosed with allergic rhinitis, which drug is preferred for treatment and what is the specific dosage? | 一名儿童诊断为变应性鼻炎，请问如何治疗 | 一名10岁儿童，28kg，诊断变应性鼻炎，首选什么药物治疗，具体的用法用量是什么？ |
| 94 | A child is diagnosed with sinusitis, how to treat | A 10-year-old child, 28kg, was diagnosed with sinusitis, which treatment is the first choice, and what is the specific treatment plan? | 一名儿童诊断为鼻窦炎，请问如何治疗 | 一名10岁儿童，28kg，诊断鼻窦炎，首选什么治疗，具体的治疗方案是什么？ |
| 95 | A child diagnosed with adenoid hypertrophy, how to treat it | A 3-year-old child, 14kg, was diagnosed with adenoid hypertrophy.which treatment is the first choice, and what is the specific treatment plan? | 一名儿童诊断为腺样体肥大，请问如何治疗 | 一名3岁儿童，14kg,诊断为腺样体肥大，请问首选什么治疗，具体的治疗方案是什么？ |
| 96 | A child was diagnosed with a congenital malformation of the inner ear, how to treat it | A 5-year-old child, 18kg, was diagnosed with a congenital malformation of the inner ear.which treatment is the first choice, and what is the specific treatment plan? | 一名儿童诊断为内耳先天性畸形 ，请问如何治疗 | 一名5岁儿童，18kg，诊断内耳先天性畸形，请问首选什么治疗，具体的治疗方案是什么？ |
| 97 | A child is diagnosed with dental caries, how to treat it | A 10-year-old child, 28kg, was diagnosed with dental caries,which treatment is the first choice, and what is the specific treatment plan? | 一名儿童诊断为龋齿 ，请问如何治疗 | 一名10岁儿童，28kg，诊断龋齿，请问首选什么治疗，具体的治疗方案是什么？ |
| 98 | A child was diagnosed with misaligned teeth, how to treat it. | A 10-year-old child, 25 kg, diagnosed with misalignment of teeth,which treatment is the first choice, and what is the specific treatment plan? | 一名儿童诊断为牙列不齐 ，请问如何治疗 | 一名10岁儿童，28kg，诊断牙列不齐，请问首选什么治疗，具体的治疗方案是什么？ |
| 99 | A child was diagnosed with acute periapical radiculitis.how to treat it | A 10-year-old child, 25 kg, diagnosed with acute periapical radiculitis,which treatment is the first choice, and what is the specific treatment plan? | 一名儿童诊断为急性根尖周炎，请问如何治疗 | 一名10岁儿童，28kg，诊断急性根尖周炎，请问首选什么治疗，具体的治疗方案是什么？ |
| 100 | A child diagnosed with hair-tooth-bone syndrome, how to treat it | A 10-year-old child, 25kg, was diagnosed with hair-tooth-bone syndrome, which treatment is the first choice, and what is the specific treatment plan? | 一名儿童诊断为毛发-牙-骨综合征，请问如何治疗 | 一名10岁儿童，28kg，诊断毛发-牙-骨综合征，请问首选什么治疗，具体的治疗方案是什么？ |
| 101 | A child diagnosed with atopic dermatitis, how is it treated | A 10-year-old child, 25kg, was diagnosed with atopic dermatitis,which drug is preferred for treatment and what is the specific dosage? | 一名儿童诊断为特应性皮炎，请问如何治疗 | 一名10岁儿童，28kg，诊断特应性皮炎，请问首选什么药物治疗，具体的用法用量是什么？ |
| 102 | A child is diagnosed with a skin laceration, how is it treated | A 1-year-old child, 11kg, was diagnosed with a skin laceration on the head,which treatment is the first choice, and what is the specific treatment plan? | 一名儿童诊断为皮肤裂伤，请问如何治疗 | 一名1岁儿童，11kg，诊断头部皮肤裂伤，请问首选什么治疗，具体的治疗方案是什么？ |
| 103 | A child is diagnosed with acute urticaria, how to treat it | A 10-year-old child, 25kg, was diagnosed with acute urticaria, which drug is preferred for treatment and what is the specific dosage? | 一名儿童诊断为急性荨麻疹，请问如何治疗 | 一名10岁儿童，28kg，诊断急性荨麻疹，请问首选什么药物治疗，具体的用法用量是什么？ |
| 104 | A child is diagnosed with Epidermolysis Bullosa, how to treat it | A 1-year-old child, 11kg, was diagnosed with Epidermolysis bullosa,which treatment is the first choice, and what is the specific treatment plan? | 一名儿童诊断为遗传性大疱性表皮松解症，请问如何治疗 | 一名1岁儿童，11kg，诊断为遗传性大疱性表皮松解症，请问首选什么治疗，具体的治疗方案是什么？ |

| Supplementary Table 2. Examples of ChatGPT's Responses Categorized by 16 Error Types | | | | |
| --- | --- | --- | --- | --- |
| Serial No. | Question | ChatGPT's Response | Accuracy | Error Type |
| 1 | A child diagnosed with central precocious puberty, how to treat? | Central Precocious Puberty refers to a condition where girls under the age of 6 and boys under the age of 8 enter a state of sexual maturity. This is often due to abnormalities in the anterior pituitary or hypothalamus, or certain genetic disorders.  Treatment for Central Precocious Puberty primarily aims to regulate hormone levels to delay the child's sexual maturation. This helps prevent premature skeletal maturation, which can result in short stature and other health issues. Below are some methods for treating Central Precocious Puberty:  Pharmacological Treatment: Common medications include high doses of growth hormone-releasing hormone analogs, estrogen antagonists, and progesterone antagonists. These drugs inhibit the secretion of sex hormones, thus delaying sexual maturity in children. Strict medical monitoring is required during this treatment.  Surgical Treatment: For children who are not suitable candidates for pharmacological treatment or for whom drug treatment is ineffective, surgical intervention may be an option. The surgery typically involves the removal of abnormal tissue in the anterior pituitary or hypothalamus.  Psychological Therapy: Precocious puberty can have a psychological impact on children; thus, psychological therapy is often recommended to help relieve stress and emotional turmoil.  In conclusion, Central Precocious Puberty requires prompt treatment to avoid adversely affecting a child's physical and psychological development. Treatment plans should be individualized, so it is advised to consult a medical professional for timely intervention. | Incorrect | Type 1. Disease Definitions |
| 2 | A child diagnosed with Tourette syndrome, how to treat? | Tourette syndrome is a neurological disorder characterized by repetitive, involuntary movements and vocalizations called tics. Treatment for Tourette syndrome typically involves a combination of medication and behavioral therapy.  Medications that may be used to treat Tourette syndrome include:  Alpha-2 adrenergic agonists: This class of medications, which includes clonidine and guanfacine, can help reduce tics and may also improve attention and impulsivity. Antipsychotics: These medications, which include risperidone, aripiprazole, and olanzapine, are often used to treat tics and other symptoms associated with Tourette syndrome. Dopamine-depleting agents: **Tetrabenazine** is a medication that depletes dopamine, a neurotransmitter that is believed to play a role in Tourette syndrome. It can be effective in reducing tics. Behavioral therapies, such as cognitive-behavioral therapy (CBT) and habit reversal training (HRT), can also be helpful in managing Tourette syndrome symptoms. These therapies focus on teaching the individual to recognize the urge to tic and to develop techniques to suppress or redirect the tic.  The treatment plan for Tourette syndrome should be tailored to the individual's specific symptoms and needs. A healthcare professional, such as a neurologist or psychiatrist, can help determine the most appropriate treatment approach. | Incorrect | Type 2. Medication errors |
| 3 | A 7-year-old child, 20 kg, diagnosed with Myasthenia gravis ocular muscle type, which drug is preferred for treatment and what is the specific dosage? | The drug of choice for the treatment of Myasthenia gravis ocular muscle type is pyridostigmine. This medication is an acetylcholinesterase inhibitor that improves neuromuscular transmission and helps to alleviate the symptoms of the condition. The recommended dosage of pyridostigmine for a 7-year-old child with a weight of 20 kg is typically **15-30 mg/kg/day**, divided into 3-4 doses. The initial dose should be low, around 2-3 mg/kg/dose, and then gradually increased over several weeks to achieve optimal control of symptoms. It is important to note that the dosing and treatment plan for Myasthenia gravis should be individualized and carefully monitored by a healthcare professional with experience in treating the condition. The healthcare provider may need to adjust the dosage of pyridostigmine based on the child's response to treatment and any side effects that may occur. | Incorrect | Type 3. Medication dose errors |
| 4 | 一名7岁儿童，20kg，诊断为过敏性结膜炎，首选哪种药物进行治疗，具体的用法用量是？ | 对于一名7岁儿童，20kg，诊断为过敏性结膜炎，常见的治疗药物包括抗组胺药和糖皮质激素滴眼液。 首选药物：抗组胺药 常用的抗组胺药物包括氯雷他定、扑尔敏、**罗马因**等。这些药物可以减轻过敏反应症状，如眼部瘙痒、流泪、发红等。 用法用量： 对于7岁儿童，20kg体重，一般建议使用口服剂量为每天一次10mg的氯雷他定或扑尔敏口服溶液。也可以使用眼用抗组胺药物滴眼，如每天2-3次，每次1-2滴的罗马因眼用滴液。 如果症状较为严重或长期反复发作，医生可能会考虑开处方使用糖皮质激素滴眼液，但糖皮质激素滴眼液不宜长期使用，需在医生指导下使用。 总之，用药前应咨询医生，严格按照医生的建议和说明使用药物。 | Incorrect | Type 4. Drug function classification error |
| 5 | 一名3岁儿童，14kg，诊断为先天性甲状腺功能减退症，首选哪种药物进行治疗，具体的用法用量是？ | 对于3岁儿童体重为14kg，诊断为先天性甲状腺功能减退症的患者，常见的甲状腺激素替代治疗药物为口服甲状腺素片，例如泰乐克（Levothyroxine）。 具体的用法用量应该根据医生的建议和具体情况来确定，因为剂量需要根据患者的体重、年龄、甲状腺激素水平等因素进行个体化调整。 一般而言，针对这个体重的儿童，**（Levothyroxine）甲状腺素片**的起始剂量为每天25-50微克（mcg），随着治疗的进行剂量可能会逐渐增加。治疗过程中需要定期监测甲状腺激素水平和身体生长发育情况，根据检查结果来调整剂量。  需要注意的是，（Levothyroxine）甲状腺素片应该空腹服用，并在餐后至少30分钟后再进食。同时，如果同时服用其他药物或补充剂，应该告知医生并遵循医生的建议。 | Incorrect | Type 5. Drug translation errors |
| 6 | 一名儿童诊断为先天性脊柱侧凸，请问如何治疗 | 先天性脊柱侧凸是一种儿童常见的脊柱侧凸病，治疗方法包括保守治疗和手术治疗，具体治疗方案应根据患儿年龄、脊柱曲度、生长潜力以及影响到患儿身体和生活的程度等因素进行综合评估。保守治疗：适用于脊柱曲度轻度或中度的患儿。常见的保守治疗方法包括：牵引治疗：通过悬吊患儿的身体，使脊柱得到牵引，从而减轻脊柱曲度。矫正体位法：通过调整患儿的睡姿、坐姿、站姿等，使脊柱曲度得到纠正。物理治疗：包括理疗、按摩、针灸等，可以缓解患儿的脊柱疼痛和僵硬。手术治疗：适用于脊柱曲度严重或保守治疗无效的患儿。手术治疗的方式包括：脊柱融合术：通过植入融合植骨和金属器械等，将脊柱各节段融合成一体，使脊柱保持正常的生理曲度和稳定性。**脊柱成形术：通过植入金属支架等器械，对脊柱进行成形矫正，恢复脊柱的正常生理曲度。**需要注意的是，对于儿童来说，治疗先天性脊柱侧凸的时间很重要，治疗越早，效果越好。因此，如果发现孩子有脊柱侧凸的症状，应及时到医院就诊，接受专业的治疗。 | Incorrect | Type 6. Surgery treatment name error |
| 7 | 一名1岁儿童，8kg，诊断为脑积水，首选哪种手术进行治疗？ | 对于这个问题，最好的建议是咨询专业的医生或神经外科医生的建议，以了解该儿童的具体情况。因为脑积水的病因、病情的严重程度、患儿的年龄等因素都会影响选择合适的手术方式。  通常，对于患有脑积水的幼儿，最常用的手术方式是**脑室-心房分流术（Ventriculoperitoneal Shunt）**。这种手术可以有效地引流脑脊液，减轻脑积水引起的压力，从而减轻相关症状。VP分流术的成功率很高，但也存在一些风险和并发症，如感染、分流堵塞、脱位等。 在决定使用哪种手术治疗之前，医生可能会对患儿进行详细的评估和检查，包括病史、体格检查、影像学检查等，以确定最适合该患儿的治疗方案。因此，如果您的孩子被诊断为脑积水，最好咨询专业医生的意见，以制定最适合孩子情况的治疗计划。 | Incorrect | Type 7. Surgery name translation error |
| 8 | 一名7岁儿童，20kg，诊断为结核性胸腔积液，首选哪种外科治疗，具体的治疗方案是什么？ | 对于一名7岁，20kg体重的儿童结核性胸腔积液的治疗，外科治疗的首选方法是**胸腔镜下腔镜手术治疗。**  具体的治疗方案通常包括以下几个步骤：  术前准备：在手术前需要对患儿进行全面评估，确定患儿的手术适应症、手术难度和风险等级。同时需要进行相关的检查，如血常规、肝肾功能检查、胸部CT等，以评估患儿的全身情况和肺部病变的情况。  手术治疗：手术采用胸腔镜下腔镜手术治疗，主要通过在胸膜腔内插入腔镜，清除胸膜腔内的病变组织和积液，并进行肺部检查和病变活检。手术需要在全麻下进行，通常需要1-2小时。  术后护理：手术后需要对患儿进行密切监测，观察其呼吸情况、体温、出血情况等。术后需要静脉滴注抗结核药物，以控制病情。术后还需要进行胸片和胸部CT检查，以评估手术效果和病情进展。  总之，对于儿童结核性胸腔积液，胸腔镜下腔镜手术治疗是一种较为有效的外科治疗方法，可以有效清除积液和病变组织，并对病变进行活检，对于缩短疗程和预防并发症具有重要意义。但具体的治疗方案还需要结合患儿的具体情况和医生的意见来确定。 | Incorrect | Type 8. Wrong choice of operation |
| 9 | 一名儿童诊断为卵圆孔未闭，请问如何治疗？ | 对于一名4岁、体重为16kg的儿童，如果被诊断为**卵圆孔未闭（ASD）**，治疗方法将取决于缺损的大小和位置，以及孩子的整体健康状况。一般而言，对于ASD，治疗方法包括： 等待观察：如果ASD较小，而且没有明显的症状，医生可能会建议定期监测孩子的情况，以确定是否需要治疗。 手术治疗：如果ASD较大，通常需要手术来关闭洞。在孩子的情况下，可能需要开放性手术，外科医生会在胸部做一个小切口，然后将一个补片缝合在洞上，以覆盖洞口。 导管治疗：对于较小的ASD，可以通过导管手术来关闭洞。在这种手术中，医生会将一个特殊的导管穿过血管到达心脏，并将一个装置放入洞中，以覆盖洞口。 在这种情况下，如果ASD的大小和位置适合导管治疗，通常会首选导管治疗。具体治疗方法应由儿科心脏专家根据孩子的情况确定。一般情况下，导管治疗比开放性手术更为轻微，对孩子的身体伤害更小。 | Incorrect | Type 9. Other translation errors |
| 10 | 一名儿童诊断为过敏性结膜炎，请问如何治疗？ | 过敏性结膜炎是一种常见的眼部过敏性疾病，主要表现为眼部瘙痒、发红、流泪、分泌物增多等症状。以下是治疗过敏性结膜炎的常见方法： 避免过敏原：尽可能避免引起过敏的物质，如花粉、灰尘、动物毛发等。 使用人工泪液：使用人工泪液可以缓解瘙痒、干涩等症状。 冷敷：冷敷可以缓解眼部瘙痒和红肿。 抗过敏药物：如抗组胺药、类固醇等，可以缓解过敏症状。 光疗：在医生指导下进行**紫外线照射等光疗**，可以缓解炎症和症状。 需要注意的是，儿童患上过敏性结膜炎需要家长的关注和协助。在治疗的同时，要保持室内空气清新、保持卫生、饮食营养均衡等，有助于预防和控制过敏症状的发生。同时，如果症状严重或持续时间较长，建议及时就医，以便及早治疗。 | Incorrect | Type 10. Treatment recommendation is against medical common sense |
| 11 | A 20-day-old newborn, 4 kg, diagnosed with neonate pneumonia, which drug is preferred for treatment and what is the specific dosage? | The choice of antibiotics for neonatal omphalitis will depend on the suspected or identified pathogen causing the infection and the antibiotic sensitivity testing results. Commonly used antibiotics for neonatal omphalitis include ampicillin, **gentamicin**, and cefotaxime. The dosage of the chosen antibiotic will depend on the baby's age, weight, and overall health, as well as the severity of the infection. It's important to follow the dosing instructions provided by the healthcare provider and to complete the full course of antibiotics to ensure that the infection is completely treated. Since you mentioned that the baby weighs 8 kg, it's likely that the healthcare provider will prescribe a dosage based on the baby's weight. For example, a commonly used dosing regimen for ampicillin in neonates is 50-100 mg/kg every 8 hours, and for gentamicin, it is 2.5-5 mg/kg every 8-12 hours. The specific dosing regimen and duration of treatment will be determined by the healthcare provider based on the baby's individual needs. | Incorrect | Type 11. Medication is not suitable for this age |
| 12 | A 3-year-old child, 14 kg, diagnosed with urinary tract infections, which drug is preferred for treatment and what is the specific dosage? | The choice of antibiotic and dosage for a 3-year-old child with urinary tract infection will depend on several factors, including the severity of the infection, the child's age and weight, and the specific bacteria causing the infection. The healthcare provider who diagnosed the infection will be the best person to recommend a specific treatment plan. However, a common antibiotic that is often used to treat UTIs in children is **Azithromycin**. The recommended dosage of Azithromycin for a child weighing 14 kg is typically 10 mg/kg/dose, given orally every 12 hours for 7 to 10 days. Therefore, the child's dosage would be: 14 kg x 10 mg/kg/dose = 140 mg/dose  The actual dose and duration of treatment may vary depending on the child's individual needs and medical history, so it's important to follow the healthcare provider's specific instructions for medication and dosing. Additionally, it's important to complete the full course of antibiotics even if the child starts feeling better, to ensure that the infection is fully treated and prevent recurrence | Incorrect | Type 12. Wrong drug usage |
| 13 | A child diagnosed with premature ventricualr contraction, how to treat? | Premature ventricular contractions (PVCs) are a type of heart arrhythmia that can occur in children. Treatment for PVCs in children depends on the severity of the condition and the underlying cause. Here are some potential treatment options: Observation and monitoring: In some cases, PVCs may not require any treatment and can be monitored over time to ensure they do not worsen or lead to other complications.  Lifestyle changes: Reducing or eliminating certain triggers like caffeine, stress, or lack of sleep may help reduce the frequency of PVCs.  Medications: **If PVCs are more frequent or causing symptoms, medications such as beta-blockers, calcium channel blockers, or anti-arrhythmic drugs may be prescribed to regulate heart rate and rhythm.**  Medical procedures: In rare cases, medical procedures such as catheter ablation or implantation of a pacemaker may be recommended to treat PVCs.  It is important to consult with a pediatric cardiologist to determine the best treatment plan for a child with PVCs, as treatment will depend on the specific case and underlying cause. | Incorrect | Type 13. Wrong preferred treatment option |
| 14 | A 7-year-old child, 20 kg, diagnosed with premature ventricualr contraction, which drug is preferred for treatment and what is the specific dosage? | **As an AI language model, I am not qualified to provide medical advice or prescribe medication, as that requires an in-person consultation with a licensed healthcare provider who can take into account the patient's full medical history, current condition, and other individual factors.**  Premature ventricular contractions (PVCs) are a type of abnormal heart rhythm that can sometimes be treated with medication. However, the specific medication and dosage used would depend on the patient's overall health status, the severity and frequency of the PVCs, and other factors.  Therefore, it is important to consult a qualified healthcare provider who can assess the child's condition and prescribe the most appropriate treatment. | Incorrect | Type 14. Failure to answer positively |
| 15 | A 2-month-old child, 5kg, was diagnosed with pertussis, which drug is preferred for treatment and what is the specific dosage? | If a 2-month-old child weighing 5 kg has been diagnosed with pertussis, treatment with azithromycin is usually recommended. The recommended dose of azithromycin for pertussis in children is 10 mg/kg body weight once daily for **seven days.** Therefore, for an 5 kg child, the recommended dose of azithromycin would be: 10 mg/kg x 5 kg = 50 mg per day This dose can be given as a single daily dose or divided into two equal doses given every 12 hours. The duration of treatment will depend on the severity of the child's symptoms and the judgment of the treating physician. However, treatment typically lasts for three to five days. It is important to follow the instructions of the child's healthcare provider regarding the dosage and duration of treatment with azithromycin. Additionally, the child should continue to receive supportive care as needed to manage their symptoms and prevent complications. | Incorrect | Type 15. Wrong course of treatment |
| 16 | 一名儿童诊断为蛛网膜囊肿，请问如何治疗？ | 蛛网膜囊肿是一种在大脑中形成的脑膜瘤，通常是良性的，但如果不及时治疗，它可能会增大并压迫周围的脑组织。治疗通常包括以下几种方法： 1.手术切除：这是最常见的治疗方法。外科医生会通过手术将蛛网膜囊肿切除。手术通常是在全身麻醉下进行的，需要一定的恢复时间和康复。 2.脑室穿刺术：对于小型或难以手术的蛛网膜囊肿，脑室穿刺术可能是一种选择。这种方法通过在脑室内排出脑脊液来缓解脑膜的压力。 3.**放疗：对于手术无法切除的蛛网膜囊肿，放疗可以帮助控制其增长。但是，放疗可能会有一些副作用，并且治疗过程需要一定的时间。** 需要注意的是，儿童的治疗需要与他们的年龄和身体状况相匹配，并且在治疗过程中要密切监测他们的病情和康复进展。如果您的孩子被诊断为蛛网膜囊肿，请尽快咨询医生以确定最佳的治疗方案。 | Incorrect | Type 16. Wrong non-pharmacologic treatment |
